# Negative affect moderates the effect of social rejection on frontal and anterior cingulate cortex activation in borderline personality disorder

**DOI:** 10.3758/s13415-019-00716-0

**Published:** 2019-06-04

**Authors:** Johannes Sebastian Wrege, Anthony Charles Ruocco, Sebastian Euler, Katrin H. Preller, Mareike Busmann, Louisa Meya, André Schmidt, Undine E. Lang, Stefan Borgwardt, Marc Walter

**Affiliations:** 1 Department of Psychiatry, University Hospital for Psychiatry Basel, Wilhelm Klein-Strasse 27, CH-4002 Basel, Switzerland; 2 Department of Psychology, University of Toronto, Toronto, Ontario Canada; 3 Neuropsychopharmacology and Brain Imaging, Department of Psychiatry, Psychotherapy and Psychosomatics, University Hospital for Psychiatry Zurich, Zurich, Switzerland

**Keywords:** Borderline personality disorder, Social exclusion, Functional magnetic resonance imaging, Negative affect, Anterior cingulate cortex, Rejection

## Abstract

Patients with borderline personality disorder (BPD) have a heightened sensitivity to social exclusion. Experimental manipulations have produced inconsistent findings and suggested that baseline negative affect (NA) might influence the experience of exclusion. We administered a standardized social exclusion protocol (Cyberball paradigm) in BPD (*n* = 39) and age-matched and sex-matched healthy controls (*n* = 29) to investigate the association of NA on social exclusion and activation in brain regions previously implicated in this paradigm. Compared with controls, patients with BPD showed higher activation during social exclusion in the anterior cingulate cortex (ACC), the medial prefrontal cortex (mPFC), and in the right precuneus. Prescan NA ratings were associated with higher brain activation in the ACC and mPFC over all conditions, and post hoc *t* tests revealed that differences between the groups were only significant when controlling for NA. Brain activation during exclusion was correlated with NA separately for each group. Only BPD patients showed a significant association of NA and exclusion related precuneus activation (*r* = .52 *p* = .001). Additionally, BPD patients experienced less feelings of belonging compared with a healthy control (HC) group during inclusion and exclusion, although they estimated their ball possessions significantly higher than did the HC. These findings suggest that baseline NA has a crucial impact on Cyberball-related brain activation. The results underscore the importance of considering levels of NA in social exclusion protocols for participants high in this trait.

**Electronic supplementary material:**

The online version of this article (10.3758/s13415-019-00716-0) contains supplementary material, which is available to authorized users.

Borderline personality disorder (BPD) is a severe psychiatric disorder that affects approximately 1% of adults (Lenzenweger, Lane, Loranger, & Kessler, 2007). Borderline personality disorder is characterized in part by a pervasive pattern of difficulties in interpersonal relationships. In particular, BPD is associated with a hypersensitivity to social rejection (Staebler et al., 2011), which has been theorized to result from a strong reactivity to interpersonal stressors (Gunderson & Lyons-Ruth, 2008). Rejection sensitivity is also thought to play a role in other characteristic symptoms of BPD, such as affective instability and impulsive behaviors (Berenson, Downey, Rafaeli, Coifman, & Paquin, 2011; Selby, Ward, & Joiner, 2010).

Sensitivity to interpersonal rejection has been studied in BPD in many ways (Lazarus, Cheavens, Festa, & Rosenthal, 2014). Among the most common experimental approaches is the “Cyberball” paradigm, which induces feelings of ostracism from two other alleged players while participating in a virtual ball-tossing game that comprises two conditions: inclusion and exclusion (Williams & Jarvis, 2006). Compared with the inclusion condition, social exclusion induces negative emotions, such as anger and sadness, in healthy participant groups (Williams, 2007). Individuals with BPD report higher negative emotions during exclusion and inclusion conditions compared with a healthy control (HC) group (Renneberg et al., 2012), and they report feeling more excluded while also perceiving that they possessed the ball less often during the inclusion condition (Staebler et al., 2011). De Panfilis, Riva, Preti, Cabrino, and Marchesi (2015) showed that BPD patients’ reported higher negative emotions during the inclusion condition decreased to a comparable level of that of HC under the condition of overinclusion. Similarly, Weinbrecht, Niedeggen, Roepke, and Renneberg (2018) revealed more perceived ostracism during the inclusion condition among BPD and social anxiety disorder (SAD) patients compared with the HC group. In contrast to SAD, only BPD patients revealed stronger need threat. Additionally, participants with BPD report higher levels of negative affect (NA) than controls prior to beginning the Cyberball paradigm (Dixon-Gordon, Gratz, Breetz, & Tull, 2013; Staebler et al., 2011), which might impact the effects of the experimental manipulation. Dixon-Gordon et al. (2013) found that pre-task NA was significantly associated with ratings of social rejection in BPD and HC after exclusion from the ball-tossing game, suggesting that baseline NA might moderate the effects of the social exclusion condition on ratings of social rejection for individuals with BPD.

According to a recent review incorporating studies of both healthy and clinical groups (Wang, Braun, & Enck, 2017), a network of brain regions has been implicated in social exclusion based on research using the Cyberball task: inferior parietal lobule (IPL), precuneus, anterior cingulate cortex (ACC), and prefrontal cortex (PFC). Cacioppo et al. (2013) suggest that anterior insula (AI) and ACC play an integrative role in producing subjective emotional experiences, but discrete areas of the ACC are thought to play different roles in detecting expectancy violations and social feedback (Bolling et al., 2011; Somerville, Heatherton, & Kelley, 2006). The ventromedial PFC (vmPFC) has frequently been tied to the generation and regulation of negative emotions (Hiser & Koenigs, 2018), and portions of the dorsolateral prefrontal cortex (dlPFC) serve as additional emotion regulation sites during Cyberball rejection (Sebastian et al., 2011). The IPL, as part of the temporo-parietal junction (TPJ), and the precuneus are areas involved in mentalizing and self-referential processes (Cavanna & Trimble, 2006; Schurz, Radua, Aichhorn, Richlan, & Perner, 2014).

Neuroimaging studies applying the Cyberball paradigm to patients with BPD have revealed biased perceptions of social inclusion and corresponding differences in brain activation. Domsalla et al. (2014) found that patients with BPD reported higher feelings of rejection during inclusion, although they did not feel more rejected than the HC group during exclusion. Imaging results indicated that patients showed higher activation than those in the HC group in dACC, mPFC, AI, and precuneus, regardless of experimental condition. These findings suggest that patients with BPD consistently engaged brain regions involved in social cognition and emotion regulation, regardless of the extent to which they were included in the social interaction, which may be reflected in their reported feelings of rejection even during inclusion. Similarly, Gutz, Renneberg, Roepke, and Niedeggen (2015) found the P3b event-related potential, which has been associated with social rejection (Polich, 2007), was elevated in patients with BPD during inclusion compared with the HC group and patients with SAD. Weinbrecht et al. (2018) recently reproduced this finding of an enhanced P3 complex during inclusion in BPD and SAD patients compared with HC, which decreased during the overinclusion condition. Overall, these findings suggest a neurophysiological basis for a biased perception of rejection during social inclusion that are reported by patients with BPD.

To our knowledge, the association between baseline NA and feelings of rejection and its neural correlates following social exclusion in BPD have not yet been investigated. If the experience of exclusion elicits strong inner tension, which lasts long into daily social contexts (Stiglmayr et al., 2005), we hypothesize that NA before scanning may have an impact not only on the subjective intensity of rejection feelings but also on the corresponding neural activation patterns in BPD. In the present study, we measured negative affect prior to administering the Cyberball paradigm, which included social inclusion and exclusion conditions, and then measured brain activation in patients with BPD and the HC group using functional magnetic resonance imaging (fMRI). We hypothesized that patients with BPD would show differential brain activation during social exclusion and inclusion compared with HC participants. We expected that they would display greater activation in brain regions involved in emotion regulation and social cognition—namely, the ACC, ventromedial and ventrolateral PFC. We also expected BPD patients to report fewer feelings of belongingness during both conditions of exclusion and inclusion. We assumed that these anticipated differences in brain activation and subjective experiences of Cyberball would be impacted by NA.

## Material and method

### Eligibility criteria

To be included in the study, patients were required to meet criteria for BPD according to the *Diagnostic and Statistical Manual of Mental Disorders–Fourth Edition–Text Revision* (DSM-IV-TR; American Psychiatric Association, 2000). Healthy control participants were not permitted to have a current or prior history of a DSM-IV Axis I or Axis II disorder. All participants were required to be at least 18 years of age and be capable of providing informed consent to participate in the study. Participants were not allowed to consume alcohol or nonalcoholic substances (including benzodiazepine but excluding nicotine) for at least 3 days prior to participation. Participants were not eligible if they reported acute psychotic symptoms. Standard exclusion criteria for MRI scanning were also applied.

### Participant characteristics

Patients with BPD (*n* = 39) were consecutively recruited from a 12-week in-patient treatment program in the Psychiatric University Hospital (UPK Basel) in Switzerland. The diagnosis of BPD was assessed using the Structured Clinical Interview for DSM-IV-TR Axis II Personality Disorders (SCID-II)—German Version (Wittchen, Zaudig, & Fydrich, 1997), which was administered by either a licensed psychiatrist or clinical psychology trainee supervised by a licensed psychiatrist. Information from the Axis I and Axis II diagnostic interviews were reviewed in a diagnostic meeting to arrive at consensus diagnoses. The HC participants (*n* = 29) were recruited using Internet advertisements on the clinic and psychology department websites. The HC participants completed the same psychiatric diagnostic interviews and questionnaires as participants with BPD. Sociodemographic and clinical characteristics of the participants (BPD and HC) are presented in Table 1. As anticipated, BPD and HC participants did not differ in age and sex; however, HC participants had more years of education and higher IQs (*p*s < .01), as assessed using the Multiple Choice Vocabulary Test–German Version (MWT-B; Lehrl, 2005).

**Table 1:** Sociodemographic and clinical date

	BPD (*n* = 39)	HC (*n* = 29)	BPD vs. HC
Female (*n*, %)	30, 76.9	25, 86.2	Fisher’s exact: *p* = .37
Male (*n*, %)	9, 23.1	4, 13.8	
Age in years (*M*, *SD*)	27.5, 8.2	25.7, 6.0	*t*(65.99) = 1.02, *p* = .31
Education in years (*M*, *SD*)	12.7, 1.9	15.9, 2.9	*t*(45.47) = −5.12, *p* < .01**
IQ (*n*, %)	97.5, 6.5	104.4, 12.0	*t*(39.94) = −2.80, *p* < .01**
Depressive disorders (*n*, %)	17, 43.6	0, 0.0	Fisher’s exact: *p* < .01**
Anxiety disorders (*n*, %)	6, 15.4	0, 0.0	Fisher’s exact: *p* < .05*
Substance-related disorders (*n*, %)	14, 35.9	0, 0.0	Fisher’s exact: *p* < .01**
Eating disorders (*n*, %)	5, 12.8	0, 0.0	Fisher’s exact: *p* = .05
Somatoform disorders (*n*, %)	2, 5.1	0, 0.0	Fisher’s exact: *p* = .49
Posttraumatic stress disorder (*n*, %)	3, 7.7	0, 0.0	Fisher’s exact: *p* = .24
Current psychotropic medication (*n*, %)	23, 59.0	0, 0.0	Fisher’s exact: *p* < .01**
Global Assessment Scale (*M*, *SD*)	42.56, 7.87	–	–
Beck Depression Inventory (*M*, *SD*)	26.40,10.97	2.38, 2.49	*t*(43.17) = 13.23, *p* < .01**
Borderline Symptom List 23 (*M*, *SD*)	1.84, 0.91	0.19, 0.19	*t*(42.30) = 11.05, *p* < .01**

*Note.* BPD = borderline personality disorder; HC = healthy control; *M* = mean; *SD* = standard deviation. Global Assessment Scale: Scores of 41–50 indicate serious symptoms (American Psychiatric Association, 2000); Beck Depression Inventory: A cut-off score of 18 indicates clinical relevance (Hautzinger, Bailer, Worall, & Keller, 1995). Borderline Symptom List 23: Validation sample of BPD patients showed *M* = 2.05, *SD* = 0.90 (Wolf et al., 2009). *p < .05. **p < .01

Table S1 in the Supplemental Material gives information of medications used in the clinical group. Baseline levels of NA were measured immediately prior to the MRI scan administering the German version of the Positive and Negative Affect Schedule (PANAS; Krohne, Egloff, Kohlmann, & Tausch, 1996). This questionnaire consists of positive (PA) and negative affect (NA) scales. Each scale consists of 10 items rated using a Likert scale ranging from 1 (*very low*) to 5 (*very strong*). The BPD patients and HC participants differed significantly in NA (BPD: *M* = 18.95, *min* = 11, *max* = 30, *SD* = 4.97; HC: *M* = 12.31, *min* = 10, *max* = 16, *SD* = 1.71), *t*(49.39) = 7.74, *p* < 0.01. (Please see Fig. S1a–b in the supplemental material for detailed information.)

### Procedures

After admission to the psychiatric treatment unit, patients were approached to assess their interest in participating in the research study. After a full description of the study, all participants provided written informed consent to participate in the study. Participants completed an MRI screening form, sociodemographic and symptom questionnaires, and semistructured psychiatric diagnostic interviews. Directly prior to the MRI scan, participants completed a urine toxicology screen, a breathalyzer test, and filled out the PANAS. Subsequently, they underwent an anatomical MRI scan and then completed the Cyberball procedure, followed by other tasks and MRI sequences not reported in the present article.

### Measures

#### Needs Threat Scale (NTS)

The NTS (Hartgerink, van Beest, Wicherts, & Williams, 2015; Williams, 2009) is a scale that is commonly used in studies employing the Cyberball procedure to measure feelings of social rejection. The NTS consists of 20 items that are rated on a 7-point Likert scale from 1 (*do not agree*) to 7 (*agree*). There are four subscales: Belonging, Self-Esteem, Control, and Meaningful Existence. In the present study, we focused on the Belonging scale, as that scale measures the extent to which participants felt that they were included in the interaction. The NTS was administered to participants retrospectively after the MRI session was completed according to how they felt after the inclusion and exclusion conditions. A subset (*n* = 23 BPD patients and *n* = 28 HC participants) of the data is also presented in Euler et al. (2018), where a compound measure consisting of the overall mean index of the NTS was computed and was found to correlate with other behavioral data from the treatment within the BPD group.

### Magnetic resonance imaging

#### Ostracism manipulation: Cyberball

**Fig. 1:**
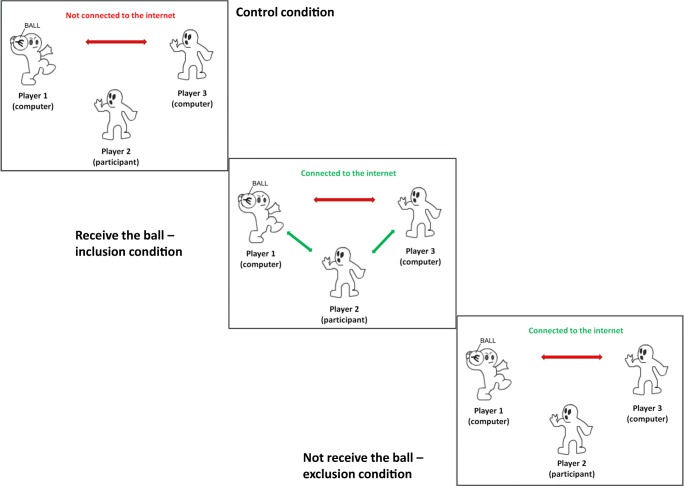
Cyberball game screen setup. In addition to the comic, participant pictures and names of other players were shown. The task consisted of three conditions (control, inclusion, exclusion). Different arrow colors represent the ball condition for the participants. Red = the participant did not receive the ball. Green = the participant received the ball. (Color figure online)

The Cyberball experimental procedure (Williams, Cheung, & Choi, 2000) was administered to participants to induce feelings of social rejection during the fMRI scan. The computer-based game consisted of three experimental conditions presented in the following fixed order: control, inclusion, and exclusion (see Fig. 1). Participants were shown a screen with three animated avatars tossing a virtual ball from one avatar to another. They were told that they are participating in a visual mentalization task and that they have to empathize with two other real-life players over the Internet; instead, the avatars were programmed to behave in a specific manner based on the experimental condition. Photographs of virtual coplayers were presented next to the avatars. Each participant’s photograph was taken before the scan, presented to them as the third participant in the game, and appeared below the participant’s avatar.

During the *control* condition, the participant was excluded by receiving a notification that the Internet was currently disconnected. They did not get any ball tosses during the control run, and the participant was asked to watch the programmed coplayers toss the ball 60 times to each other. During the *inclusion* condition, each player received the ball 30 times, 90 ball tosses between all three players in total. During the *exclusion* condition, the participant received five ball tosses at the beginning of the run, and then the preprogrammed players excluded the participant until a total of 60 ball tosses was reached. The order of throws and hesitations (intertrial interval) to throw the ball was randomized.

Participants were asked to evaluate the credibility of the cover story of playing with real human players using a 5-point Likert scale ranging from 1 (*very unlikely*) to 5 (*very likely*) for the inclusion (BPD: *M* = 3.19, *SD* = 1.32; HC: *M* = 3.36, *SD* = 1.14) and exclusion (BPD: *M* = 2.64, *SD* = 1.36; HC: *M* = 3.14, *SD* = 1.25) conditions. There was a significant Condition × Group interaction, *F*(1, 66) = 1.91, *p* = .03. There was no group effect for both conditions: inclusion, *F*(1, 67) = 0.30, *p* = .58; exclusion, *F*(1, 67) = 2.45, *p* = .12.There was a significant condition effect in the BPD group. *F*(1, 38) = 8.59, *p* < .01, but not in the HC group, *F*(1, 28) = 2.93, *p* = .10.

#### FMRI acquisition

Participants were scanned using a 3T MRI system (Siemens Magnetom Prisma, Erlangen, Germany) and a 20-channel phased-array radio frequency head coil. During the Cyberball task, T2*-weighted echo-planar imaging (EPI) data were acquired with the following parameters: interleaved acquisition mode, 39 axial slices of 3-mm thickness, 0.5-mm interslice gap, field of view 228 × 228 mm^2^ and an in-plane resolution of 3 × 3 mm^2^. The repetition time was 2.5 s, the echo time was 30 ms, and the bandwidth was set to 2350 Hz/pixel. The total run time for all three Cyberball sessions was a maximum of 11 minutes and 30 seconds, yielding a maximum total of 280 volumes (depending on the individual’s speed at completing the task). Each session started with the acquisition of three dummy scan volumes to ensure signal stabilization. Participants selected which player to toss the ball toward using an MRI-compatible response box.

#### Preprocessing

FMRI data were processed and analyzed using SPM-12 (Welcome Department of Cognitive Neurology, London, UK; Friston (2014)). Preprocessing of fMRI images followed standard procedures: slice time correction and realignment and unwarping to spatially correct for head motion and distortions. A field map distortion correction was also performed to increase the quality of EPI images. Field map correction detects the signal intensity dropout due to magnetic field inhomogeneity. During coregistration, structural and functional images were overlaid, followed by normalization of the image to common Montreal Neurological Institute (MNI) space. The last step was smoothing with a Gaussian kernel with full width at half of maximum (FWHM) of 6 mm. Smoothing enables averaging across participants and increases signal-to-noise ratio. All EPI images were manually checked for gross motion, with a maximum of 2-mm translation and 2° rotation between volumes allowed. Low quality volumes were replaced by the mean volume of the two adjacent volumes. Seven participants (*n* = 6 BPD, *n* = 1 HC) were removed from further analysis due to movement artifacts and image quality. Furthermore, six realignment parameters were entered in the design matrix. A high-pass filter of 128 seconds was applied.

#### First-level analysis

Onset times of ball toss events in the task were recorded. Events of interest (described below) were convolved with a canonical hemodynamic response function. The first three scan volumes taken during a delay prior to the start of experimental runs (“dummy scan volumes”) were discarded. Events taking place after the connecting screen (equal in each condition) were recorded, according to the point in time at which the screen differed regarding whether the ball was thrown to a coplayer or the participant. For each condition (control, inclusion, exclusion), the onset times of ball throws for each player were extracted using R and included in SPM12 analyses as follows: participant “received the ball” and participant “did not receive the ball.”

First-level analyses were carried out using the following regressors: (a) ball tosses by other players to each other during the control condition (i.e., watching game without participating); (b) receiving ball tosses during the inclusion condition; and (c) not receiving ball tosses during the exclusion condition (see Fig. 1).

#### Second-level analyses and statistical plan

We performed a 2 × 3 full-factorial design in SPM with the first factor *group* (BPD, HC) and the second factor *condition* (control, inclusion, exclusion). *Z*-normalized values of NA as well as of four additional covariates of no interest where included in the design matrix to control for their potential association with the blood-oxygen-level-dependent (BOLD) signal: age, sex, medication, IQ. As the full factorial model does not provide information about the direction of possible between-groups effects, we also calculated two-sample *t* tests of the significant main contrast for both directions. The effect of NA was tested over both groups (*N* = 68) on a correlational basis within the full factorial model, and between the two groups applying the independent *t* test with and without NA as a covariate of no interest. In order to yield more information about the association between functional imaging and behavioral data, a Spearman rank-order correlation coefficient was calculated, which was chosen to account for potential outliers. To do this, we obtained raw beta values from the revealed task-related significant clusters of exclusion. We applied a 5-mm sphere around the peak voxel of each cluster. All correlations have been corrected for the false discovery rate according to the method of Benjamini and Hochberg (1995).

We used Pickatlas for the anatomical localization, and *p* = .001 cluster forming threshold was applied in all analyses. The results of whole-brain analysis of peak level of *p* < .001 after post hoc family-wise error rate (FWE) correction and a customized minimum of voxels according to the FWE-corrected cluster size are reported. We employed a standard family-wise error rate of *p* < .05 at the cluster level.

#### Second-level contrasts

The overall brain activation difference according to the Cyberball manipulation (within group effects) were examined with the main effect of condition of the full-factorial design. Effects of differential activation between the groups were examined with three interaction terms, as described in the following approaches.

First, we calculated the interaction between *groups* (BPD, HC) and the *exclusion contrast* (did not receive the ball during the exclusion condition minus receive the ball during the inclusion condition). The definition of *exclusion contrast* was consistent with prior research testing differences in exclusion processing after social inclusion. Our event-related design allowed us to modulate the five onsets of ball tosses during exclusion condition at the first level when the participants received the ball, and these five onsets were then skipped in the second-level exclusion contrast. Vice versa, we subtracted only volumes during the inclusion condition with onsets when the participant received the ball.

Second, we calculated the interaction between *groups* (BPD, HC) and the *control contrast* (did not receive the ball during the exclusion condition minus the control condition where participants never received the ball). We applied the same Cyberball task design as Preller et al. (2016), which included the control condition (as described above) and the standard exclusion condition. Preller et al. (2016) defined the control condition as “implicit” exclusion because the participant is socially excluded but was told that they are not part of the game, therefore lacking a social reason for the exclusion. In contrast, during the “explicit” exclusion, the participant is explicitly excluded by the coplayers, but not told that they will be excluded from the game. This contrast was defined to test differences of social exclusion processing compared with baseline.

The third interaction was calculated between *groups* (BPD, HC) and the *inclusion contrast* (receive the ball during the inclusion condition minus not receiving the ball during the control condition). We were also interested in potentially differing patterns between the groups (BPD vs. HC) with respect to the inclusion condition in contrast to baseline (control condition). This contrast was intended to isolate neural activity during the inclusion condition associated with potential feelings of rejection building on prior research showing that patients with BPD report feelings of higher social rejection (Renneberg et al., 2012) as well as accompanied alterations in neural activations (Domsalla et al., 2014; Gutz et al., 2015).

To control for potentially moderating effects of NA on the above interaction terms, NA was included in all design models as a covariate of no interest.

## Results

### Self-report

During the inclusion condition, participants received one-third (30 throws) of ball tosses, whereas in the exclusion condition they received five out of 60 tosses (8.3%). Table 2 depicts the subjective ratings of the participants. We computed a 2 × 2 ANOVA of the ball-possessing data with a factor *group* (BPD, HC) and a factor *condition* (inclusion, exclusion condition). According to the task design, we found a significant condition effect, *F*(66, 1) = 245.01, *p* < .01, with higher ratings during inclusion compared with exclusion condition. The BPD patients showed higher estimations of ball possessions than HC participants did, which was shown by a significant group effect, *F*(66, 1) = 6.27, *p* = .02. The interaction between group and condition was not significant, *F*(66, 1) = 3.91, *p* = .05. Compared with the preset ball possessions by the setup, on average, both groups overestimated their ball possessions.

**Table 2:** Self-report data of Cyberball: Ratings, ball possessions, and NTS belonging scale

	BPD (*n* = 39)		HC (*n* = 29)	
	Inclusion	Exclusion	Inclusion	Exclusion
Ball possessions (*M*, *SD*)	50.33, 21.76	13.97, 11.01	38.90, 12.45	10.69, 7.04
Belonging NTS (*M*, *SD*)	3.62, 0.64	3.28, 0.79	4.22, 0.46	3.67, 0.48

*Note.* BPD = borderline personality disorder; HC = healthy control; *M* = mean; *SD* = standard deviation; NTS = Needs Threat Scale

With respect to feelings of belongingness measured with the NTS, we also found a significant condition effect, *F*(66, 1) = 27.2, *p* < .01, with decreasing belongingness ratings during exclusion condition according to the Cyberball manipulation. The HC participants felt a greater sense of belonging than the BPD patients during inclusion and exclusion, which was indicated by a significant group effect, *F*(66, 1) = 15.12, *p* < .01. We found no interaction effect between groups and conditions, *F*(66, 1) = 1.66, *p* = .2.

As belongingness ratings of the NTS and estimations of ball possessions showed a significant group effect, we correlated them with states of NA prior to the Cyberball experiment for each group separately. We found no significant correlation.

### Imaging

#### Main effect of group

**Fig. 2:**
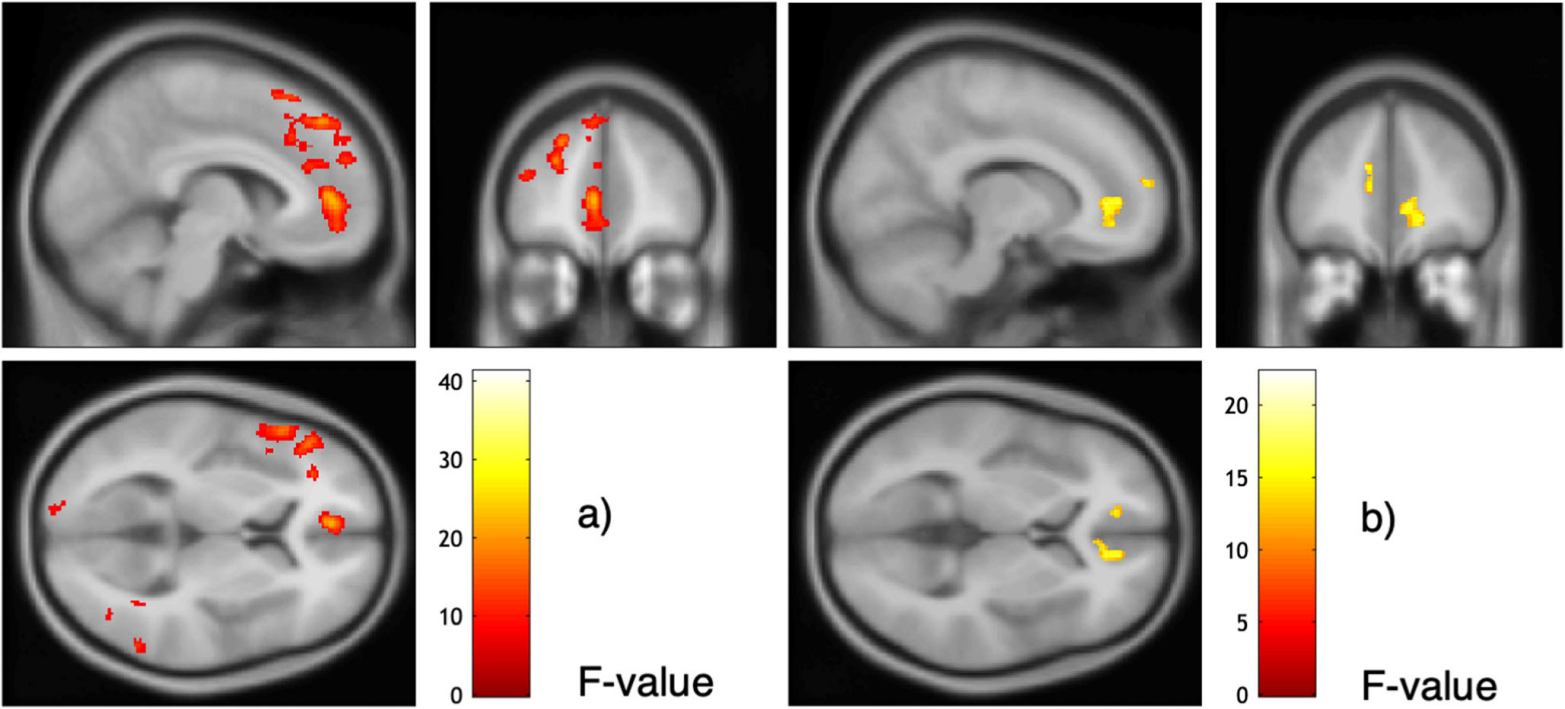
Main effects of ball-tossing condition and group. **a** Main effect of ball-tossing condition (control, inclusion, exclusion). Extended threshold: k/FWE_cluster_ = 273. **b** Main effect of group (BPD, HC). Extended threshold: k/FWE_cluster_=233

The whole-brain analysis revealed three clusters of higher activation in BPD patients compared with HC participants: the left ACC extending into the left medial superior frontal gyrus (lACC/mSFG; *p*_FWEcluster_ = .012; *F* = 22.27), the right superior and middle frontal gyri (rSFG/MFG; *p*_FWEcluster_ = .004; *F* = 18.80), and the right ACC extending into the right medial superior frontal gyrus (rACC/mSFG; *p*_FWEcluster_ = .001; *F* = 17.50; see Fig. 2; Table S3a in the Supplemental Material).

#### Main effect of task condition

A main effect of condition (control, inclusion, exclusion) of the Cyberball task among all *N* = 68 participants was associated with 13 clusters of brain activation (see Fig. 2; Table S3b in the Supplemental Material). Clusters were located in the left middle frontal gyrus (lMFG; *p*_FWEcluster_ < .001; *F* = 41.12), left angular gyrus (lAG; *p*_FWEcluster_ < .001; *F* = 39.35), the right cerebellum (*p*_FWEcluster_ < .001, *F* = 34.94), left middle temporal gyrus (lMTG; *p*_FWEcluster_ < .001; *F* = 31.51), left ACC (lACC; *p*_FWEcluster_ < .001; *F* = 21.08), right supramarginal gyrus (rSMG; *p*_FWEcluster_ < .001; *F* = 21.24), left lingual gyrus (lLG; *p*_FWEcluster_ = .001; *F* = 20.46), right opercular part of the inferior frontal gyrus (rOFC; *p*_FWEcluster_ < .001; *F* = 19.76), right fusiform gyrus (rFG; *p*_FWEcluster_ < .001; *F* = 19.68), right precuneus (rPrC; *p*_FWEcluster_ = .002; *F* = 17.64), left postcentral gyrus (lPC; *p*_FWEcluster_ < .001; *F* = 17.31), right middle frontal gyrus (rMFG; *p*_FWEcluster_ < .001; *F* = 15.49), and right superior parietal lobule (rSPL, *p*_FWEcluster_ = .001; *F* = 11.49).

#### Interaction effect of group and ball-tossing condition

**Fig. 3:**
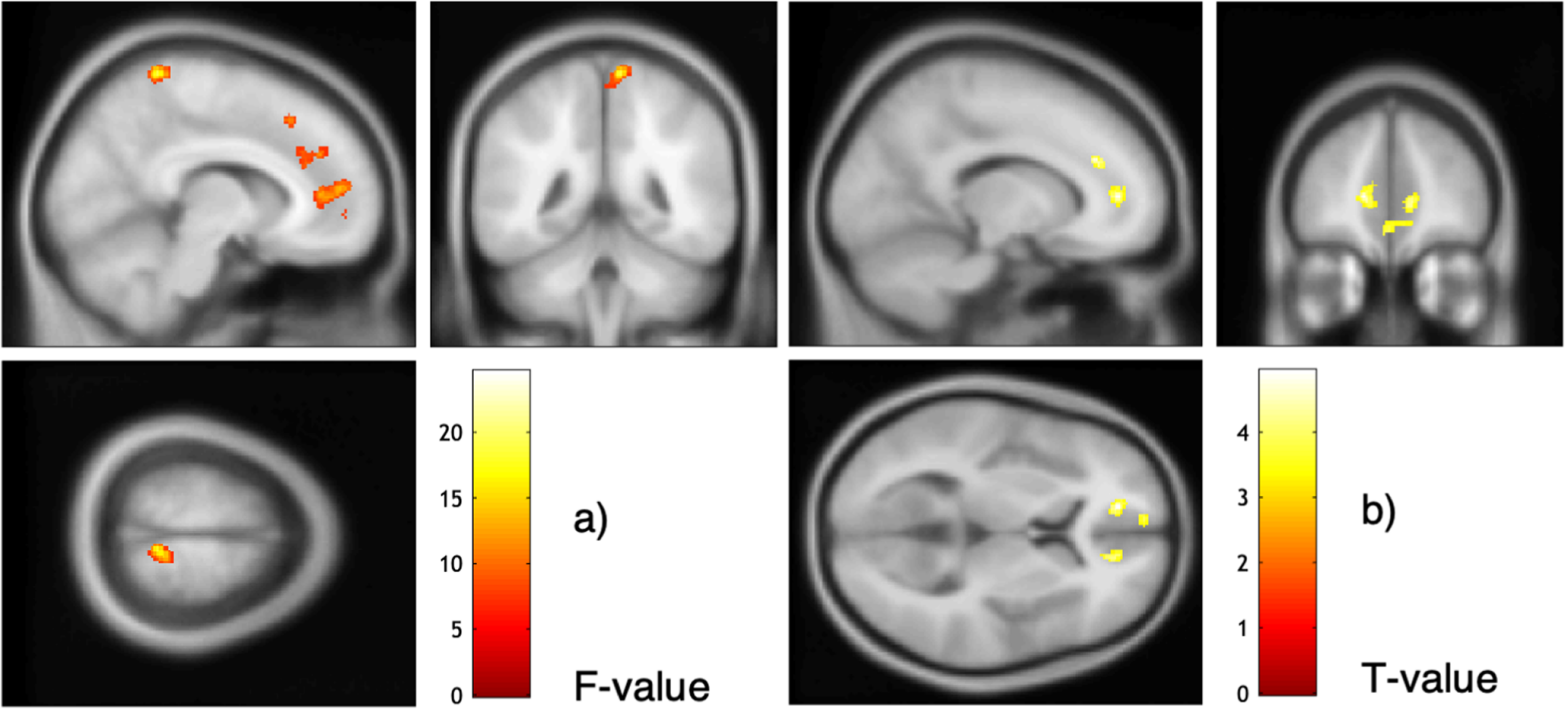
**a** Interaction effect of group (BPD, HC) and ball-tossing condition (control, inclusion, exclusion). Extended threshold: k/FWE_cluster_ = 205. **b** The *t* test (BPD > HC) of the exclusion contrast (exclusion > inclusion). Extended threshold: k/FWE_cluster_ = 336

The interaction of *group* and *ball tossing* condition revealed eight significant clusters (see Fig. 3). The clusters were located in the left ACC extending into the right medial frontal and left medial superior frontal gyrus (lACC/mFG/mSFG; *p*_FWEcluster_ < .001; *F* = 24.58), in the left superior frontal, middle frontal, and medial frontal gyri (lSFG/MFG/mFG, *p*_FWEcluster_ = .001; *F* = 18.71), in the right precuneus (rPreC, *p*_FWEcluster_ = .004; *F* = 18.57), in the right medial frontal gyrus extending into the ACC and superior frontal gyrus (rmFG/ACC/SFG); *p*_FWEcluster_ < .001; *F* = 18.46), in a cluster comprising the right superior and middle temporal gyri extending into the posterior insula (rSTG/MTG/PI; *p*_FWEcluster_ < .001; *F* = 15.14), in the left SFG/MFG/mFG (*p*_FWEcluster_ = .005; *F* = 13.10), and two clusters in the left (*p*_FWEcluster_ = .006; *F* = 12.30) and right (*p*_FWEcluster_ = .013; *F* = 11.97) cerebellum (see Table S3c in the Supplemental Material).

As the interaction between groups and the ball-tossing condition revealed significant clusters, we also calculated post hoc *t* tests in both directions, with NA as a covariate of no interest to specifically test for the direction of the effect. Whereas the *t* contrast of HC > BPD revealed no suprathreshold activation, we found three clusters of higher activation in BPD compared with HC in the exclusion contrast. Clusters were located in the medial segment of the left superior frontal gyrus and ACC (lSFG/ACC; *p*_FWEcluster_ = .004; *t* = 4.93), in the right superior frontal and middle frontal gyri extending to the frontal pole (rSFG/MFG; *p*_FWEcluster_ < .001; *t* = 4.73), and in the right ACC and medial segment of the superior frontal gyrus extending into the left ACC (rACC/SFG; *p*_FWEcluster_ < .001; *t* = 4.62; see Table S3d in the Supplemental Material). When NA was not implemented as a covariate, the *t* test was not significant.

#### Effect of negative affect as a covariate of the imaging data

We were specifically interested in the effect of NA on exclusion-related brain activation. Therefore, beta values of the significant brain activation during exclusion in the interaction between the groups and ball-tossing condition were correlated with NA states. Correlations were calculated separately for each group (BPD, HC). We skipped significant clusters in the cerebellum and correlated the brain activation of the remaining six cortical clusters (see Table S3c in the Supplemental Material) as follows: in the left ACC, left superior frontal gyrus, right precuneus, right ACC, right superior temporal gyrus, and in the left superior frontal gyrus. After correction for the false discovery rate according to Benjamini and Hochberg (1995), we found a significant correlation between NA and the brain activation in the right precuneus in the BPD group (*r* = .52, *p* = .001, q* < 0.008). The correlation between brain activation in the right precuneus and NA in the HC group (*r* = .48, *p* = .009, q* < .008) did not withstand the correction for multiple testing.

**Fig. 4:**
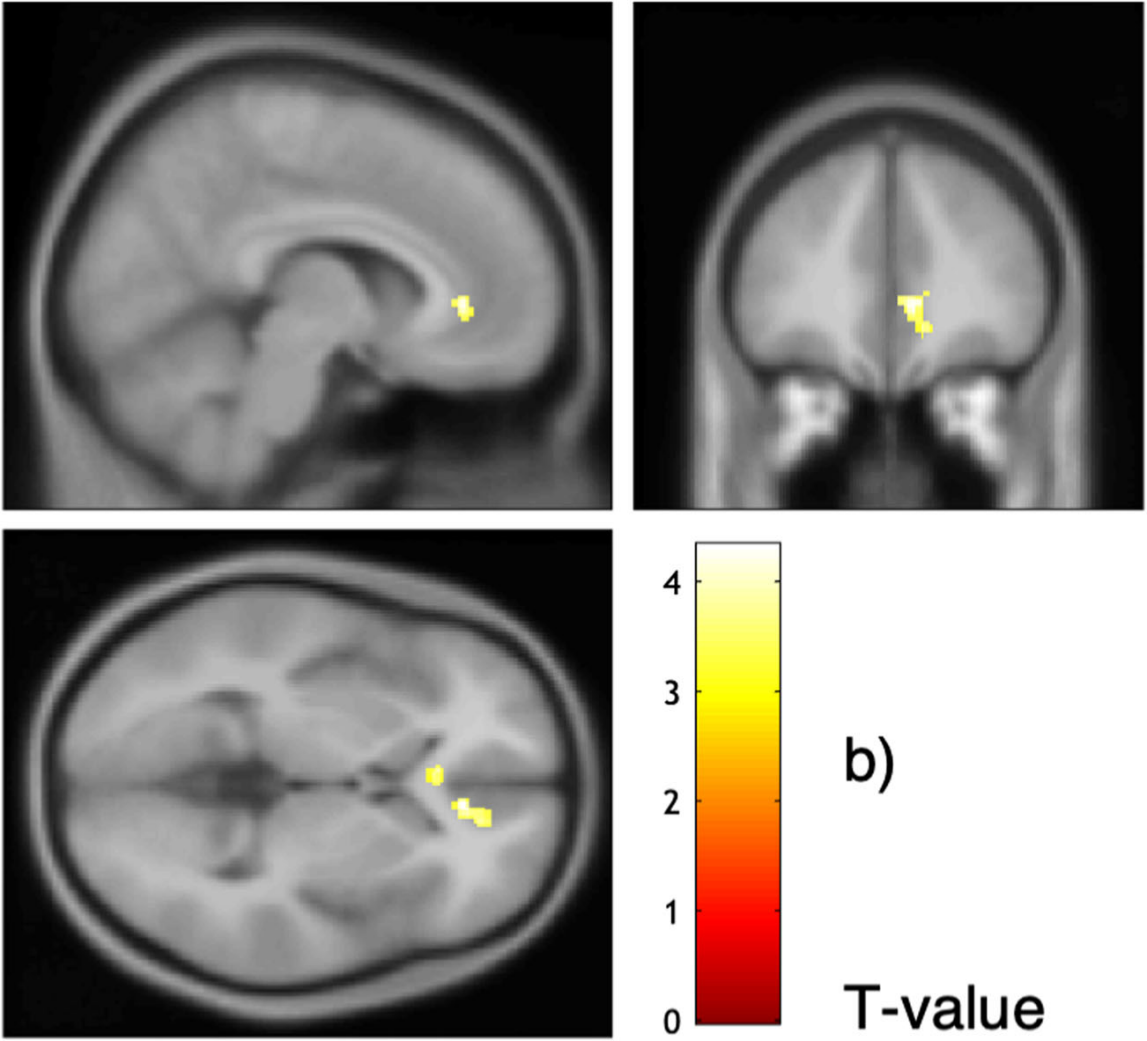
Correlation of negative affect (NA) and brain activation. Simple effect of NA in the full factorial model. Extended threshold: k/FWE_cluster_ = 482

The NA-related brain activation was analyzed within the full factorial model by defining a *t* contrast of interest where all groups, conditions, and covariates of no interest (age, sex, medication, and IQ) were set to zero and NA was set to 1. This analysis revealed a cluster comprising the ACC, medial frontal gyrus, as well as the superior and middle frontal gyri (ACC/mFG/SFG/MFG; *p*_FWEcluster_ < .001; *t* = 4.32; see Table S3e in the Supplemental Material). The higher the NA before the scan was, the higher was the brain activation in that cluster. The activation of NA-relevant brain areas (individual beta values) did not correlate with subjective ratings of belongingness (Fig. 4).

## Discussion

The aim of this study was to investigate the impact of state NA prior to Cyberball on feelings of exclusion and associated brain activity during the task. Independent of prescan NA, we expected BPD patients to show higher brain activation compared with HC participants during inclusion and exclusion. Replicating prior research, we found higher activation in BPD patients during exclusion in the ACC, medial PFC (superior and middle frontal gyri), right SMA, right precuneus, and right temporal gyri including the insula.

### Impact of negative affect on Cyberball

We expected that differences in brain activity would be moderated by NA, and that NA would also affect the subjective experience during Cyberball. Our behavioral data indicate that BPD patients experienced less feelings of belonging compared with HC participants. In contrast, they estimated retrospectively significantly more ball possessions than HC participants did. As there were significant group differences in these ratings, we correlated NA with subjective ratings of Cyberball for each group separately, and found no significant impact. However, our results are limited in their validity by our setup. We measured the behavioral data before and after the total scan session. Differences of NA after each ball-tossing condition were measured retrospectively. The NA might have changed throughout the task.

With respect to the imaging data, our results showed that NA correlated positively with brain activation during Cyberball in areas of the limbic system and frontal lobe, bilaterally, although more pronounced in the right side. This NA-related cluster overlaps with regions associated with the significant interaction between groups and ball-tossing condition: in the ACC, and in the superior and middle frontal gyri. Ventromedial prefrontal cortex (ACC/vmPFC) activity has been associated with behavioral disinhibition in BPD with respect to negative emotions also without an exclusion context (Silbersweig et al., 2007). Therefore, this stronger engagement in our patient group, independent of actual exclusion relatedness, may also be partly induced by perceived interactions with others or merely by individual differences in NA. Our full factorial design analyses controlled for NA as a regressor of no interest. Additionally, post hoc *t* tests indicated the direction of this interaction effect, revealing that BPD patients had higher brain activation than HC participants only when controlling for NA. Hence, NA had a significant overlapping impact on the exclusion-related brain activation.

We do not have information about possible changes in NA during the experiment. It may be possible that NA decreases during any condition of the experiment. In fact, postscan NA was lower compared with prescan values, which may indicate that patients dissociated during the perceived social encounter. The distribution of NA values was bimodal with little overlap between HC and BPD patients, and the average sum scores in the clinical group were significantly higher than in HC (see Fig. S1 in the Supplemental Material). The HC participants displayed a flat distribution of NA measures, but they still followed a normal distribution. Negative aspect is a clinical characteristic of BPD, and one can argue that NA functions as a discriminating (e.g., group classifying) criteria between the two participant groups included in this study. As the decrease in NA during the Cyberball task was observed in both participant groups, our data suggest that future imaging studies should control for prescan NA as a moderator variable.

### Neural activation associated with biased experience during Cyberball

As described previously, BPD patients demonstrate a biased experience of social inclusion during Cyberball (Staebler et al., 2011). Subjective experiences of BPD patients appeared to be biased in the present study with respect to belongingness ratings and gauges of ball possessions. All participants (patients and HC participants) felt similarly excluded during the exclusion condition, but HC participants felt socially more included than BPD patients, represented by our significant main effect of group of the belongingness scale. Even though we found no significant interaction effect, belongingness ratings of BPD patients during the inclusion condition were comparable to HC participants during the exclusion condition. This is in line with the findings of De Panfilis et al. (2015), in which BPD patients reported lower feelings of social connection than controls in any experimental condition of the Cyberball task. The results were interpreted to suggest that individuals with idealized interpersonal expectations in BPD may prevent the experience of real social connection. This was seen even when BPD patients were overincluded.

Regarding our main experimental manipulation (ball possessions), compared with HC participants, BPD patients showed significantly higher estimations of ball possessions during the inclusion and exclusion conditions. However, compared with the preset and fixed experimental condition, all participants, on average, overestimated their ball possessions. The BPD patients’ significantly higher estimations may have been due to the BPD group having significantly fewer years of education in our sample. We checked the experimental design and asked for the subjective plausibility of the cover story, too. As there were no differences between the groups, it indicates that the task worked comparably in patients and HC participants. Another explanation for the overestimations of ball possessions in BPD patients may be that patients focused less on the task. Interestingly, even though BPD patients overestimated ball possessions, they felt a weaker sense of belonging during the inclusion condition. De Panfilis et al. (2015), by applying a modified Cyberball task, showed that extreme overinclusion reduces negative emotions in BPD patients to levels comparable with HC participants, but not the subjective experience of social connectedness.

The subjective experience of Cyberball in BPD patients is accompanied by neural differences during both inclusion and exclusion conditions in the prefrontal cortical midline structures, the precuneus, and in the insula (see Introduction). The strength of our study is (a) a relatively high number of participants with BPD in (b) a stable, but severe clinical status (1 week hospitalized), and (c) in contrast to earlier studies, we applied an event-related design that allowed us a more precise analysis for the inclusion and exclusion conditions. During the inclusion condition, we were able to include only trials with onsets when participants received the ball, and during exclusion we deleted the trials in the beginning, when the participants were still included. The full-factorial analysis design we applied was controlled for age, sex, verbal IQ, and for medication on a dichotomous level (yes/no), but not for depressive symptoms. A recent study by Malejko et al. (2018) attempted to disentangle Cyberball-related effects in BPD from medication effects and depressive symptoms by contrasting neural activation of BPD patients with two control groups of HC and depressed patients. Potentially BPD-specific alterations during the Cyberball inclusion condition were found for the dorsomedial PFC and the PCC, which also correlated with severity of BPD symptoms. This overlaps with our main finding during exclusion networking of stronger engagement in the mPFC.

Domsalla et al. (2014) found higher BOLD signal among BPD patients during inclusion and exclusion in dACC, mPFC, AI, and in the precuneus. AI and precuneus activation was not modulated by the experimental conditions in the BPD group, and the authors interpreted this pattern of activation as neural correlates of hypermentalization in a social encounter with objectively absent rejection cues. We also found significantly higher activation in BPD patients in similar areas, including the precuneus. Our main effect group revealed higher activation in the ACC, the medial superior and middle frontal gyri of both hemispheres, and our interaction effect of group and ball-tossing condition revealed additional activation sites in the right precuneus and right temporal gyri including the posterior insula. Our post hoc *t* tests revealed that these significant activations were found in the BPD group. We correlated the brain activation of these areas during exclusion with NA, and found that the brain activation in the right precuneus was significantly influenced by NA, but only in BPD patients and not in HC participants. In other words, in contrast to Domsalla et al. (2014), we did observe ball-tossing-condition-dependent alterations in BPD patients, and, especially, right precuneus activation during exclusion was modulated by prescan NA. However, besides differences in analysis approach, this comparison with the study by Domsalla et al. (2014) is limited, given their block design and additional painful temperature stimuli. The patient samples differed too, with Domsalla et al. (2014) having examined clinical, stable outpatients without medication, in contrast to hospitalized patients with 59% having stable medication in our study.

### ACC and mPFC alteration in BPD patients during Cyberball exclusion

The main effect of condition in our study revealed typical network activation as described in prior Cyberball-imaging studies (see Introduction). Cortical midline structures (dorsal ACC, precuneus) and lateral regions (dlPFC, anterior insula and temporal gyri), especially, have repeatedly been reported to be differentially activated in several clinical groups during Cyberball (Wang et al., 2017). Dorsal ACC activation aligns with the subjective detection of exclusion and the experience of social pain, whereas ventrolateral PFC activations are associated with its regulation (Eisenberger, 2012; Kawamoto et al., 2012). The model adequacy of Cyberball in BPD and its clinical moderators have been questioned before (Kawamoto, Ura, & Nittono, 2015). Social exclusion network models are based on findings in HC participants (Cacioppo et al., 2013), while studies in BPD patients found discrepancies between different populations (Brown et al., 2017). FMRI studies at the whole-brain level found mostly prefrontal cortical activation during exclusion, but these patterns were also affected by different factors (e.g., self-esteem, gene polymorphisms, addiction; for review, see Eisenberger, 2013; Wang et al., 2017).

Compared with HC, BPD patients in our sample exhibited stronger engagement of the genual ACC and medial PFC during exclusion. Besides the overlap with NA-related engagement in the ACC/mPFC, ventral midline structures are interpreted as being responsive to social feedback and active during social cognitive and self-evaluative processes (Somerville et al., 2006; Vijayakumar, Cheng, & Pfeifer, 2017). The medial PFC also may represent regulatory functions of negative affect via connections to the ACC and amygdala (Wang et al., 2017). In contrast, activation in the ventromedial PFC and orbitofrontal cortex (OFC) has been found to be reduced during the presentation of negative stimuli (van Zutphen, Siep, Jacob, Goebel, & Arntz, 2015). Exclusion-related higher prefrontal brain activation in BPD patients also extended into the frontal pole. The frontal pole has been found to be more engaged in BPD patients during social exclusion (Ruocco et al., 2010), and this may reflect difficulties associated with self–other differentiation in BPD patients (Beeney, Hallquist, Ellison, & Levy, 2016).

Critics of the Cyberball procedure believe the dACC may correlate more directly with expectancy violation rather than with social pain (Bolling et al., 2011). A meta-analysis on fMRI designs in social pain could show that subdivisions of ACC are activated differently and that the Cyberball manipulation showed significantly less dACC activation than all other studies of social pain (Rotge et al., 2014). Specifically, self-reported distress and the emotional value of exclusion may engage more anterior regions of the ACC, the subgenual and pregenual ACC in particular. Our results are in line with Rotge et al. (2014), as BPD patients in our sample showed stronger engagement compared with HC participants in the genual and not dorsal ACC. Additionally, our data underscore the importance of self-reported distress prior to the Cyberball manipulation as a moderator of the brain activation.

### Limitations

A major limitation of the present study is that the timing of changes in NA associated with the completion of the Cybertall task could not be addressed, as the order of the conditions was not randomized in the Cyberball paradigm, and the degree to which the MRI acted as a stressor was not controlled for. As such, we were also not able to consider the effects of changes in NA after each condition. When NA is already high to start with, patients’ coping strategies during social encounters may decompensate and, as a possible result, feelings of belongingness are limited, thus limiting the validity of the task. Since moderation of the effect of this social task is very probable already in HC participants (Hartgerink et al., 2015), this may be especially important in BPD patients where social disability is relevant. We propose NA as an important moderator on the behavioral and neural level. The extent to which BOLD-signal differences observed in our study may be specific to the paradigm requires further investigation.

Our clinical group can be characterized as severe and in many cases as in an acute emotional state, but since they were hospitalized, they often show broader emotional disturbances additional to mere social-emotional distress. They had depressive symptoms and other psychiatric comorbidities (see Table 1), and were medicated. The cross-sectional design of the present study also limits analyses of stability and treatment effects of this in-patient population on social exclusion processing. Future projects employing longitudinal designs may reveal information on mechanisms of changeability and aid in differentiating BPD-specific biomarkers at the neural level.

### Conclusion

The aim of this study was to gain a deeper insight into the processing of social exclusion and how these processes relate to pretest affective states in patients with BPD. Although the impact of NA warrants further research, we could show that elevated NA before the task influenced imaging effects of the Cyberball manipulation in our sample. Our data support the hypothesis that states NA prior to task participation acts as a confounder of task-related brain activity. Post hoc *t* tests to reveal the direction of our *F* test results were rendered significant only when controlling for NA. Higher activation in BPD patients compared with HC participants were found in clusters comprising the cortical midline structures gACC/mPFC/rPreC, and activation in the precuneus was correlated with prescan NA. Our study suggests that elevated state NA should be accounted for in neuroimaging-based Cyberball designs. Long-lasting individual tension level is a key target in the treatment of BPD, and preexperimental tension level can lead to different within-group as well as between-group alterations in imaging data of social exclusion.

## Electronic supplementary material

ESM 1 (DOCX 89 kb)
