# Supplementary material for: Negative affect moderates the effect of social rejection on frontal and anterior cingulate cortex activation in borderline personality disorder
Source: Cogn Affect Behav Neurosci. 2019 Jun 4;19(5):1273–85. doi: 10.3758/s13415-019-00716-0 (PMC6785570; doi:10.3758/s13415-019-00716-0)
Supplement: Supplementary file 1 — (DOCX 89 kb) [file 13415_2019_716_MOESM1_ESM.docx]

**SI**

**Tables**

Table S1

*Medication list*

| N = 39 BPD patients (in %) |  | |
| --- | --- | --- |
| antidepressants | n = 16 | (41%) |
| antipsychotics | n = 8 | (20.5%) |
| mood stabilizer / antiepileptics | n = 6 | (15.4%) |
| other | n = 5 | (12.8%) |
| total | n = 24 | (61.5%) |

| Table S2 | | | | | |
| --- | --- | --- | --- | --- | --- |
| *Descriptive Data and mixed analysis of variance of the Need Threat Scale (NTS)* | | | | | |
| NTS subscale | Inclusion | Exclusion | Interaction effect | Condition effect | Group effect |
|  | *M*, *SD* | *M*, *SD* |  |  |  |
| Belonging | | |  | | |
| BPD | 3.62, 0.64 | 3.28, 0.79 | *F*(1, 66) = 1.66,  *p* = 0.20 | *F*(1, 66) = 27.20,  *p* < 0.01** | *F*(1, 66) = 15.12,  *p* < 0.01** |
| HC | 4.22, 0.46 | 3.67, 0.48 |  |  |  |
| Self-esteem | | |  | | |
| BPD | 4.29, 0.58 | 3.68, 1.03 | *F*(1, 66) = 1.98,  *p* = 0.16 | *F*(1, 66) = 34.54,  *p* < 0.01** | *F*(1, 66) = 15.75,  *p* < 0.01** |
| HC | 4.76, 0.33 | 4.39, 0.55 |  |  |  |
| Control | | |  | | |
| BPD | 2.85, 0.68 | 2.69, 1.08 | *F*(1, 66) = 0.10,  *p* = 0.76 | *F*(1, 66) = 3.74  *p =* 0.06 | *F*(1, 66) = 2.10  *p* = 0.15 |
| HC | 3.16, 0.80 | 2.95, 0.90 |  |  |  |
| Meaningful existence | | |  | | |
| BPD | 3.82, 0.84 | 2.98, 0.88 | *F*(1, 66) = 0.01,  *p* = 0.93 | *F*(1, 66) = 91.01  *p* < 0.01** | *F*(1, 66) = 2.97  *p* = 0.09 |
| HC | 4.11, 0.61 | 3.29, 0.75 |  |  |  |

*Note.* A lower NTS-mean indicates a higher need threat; BPD *n* = 39, HC: *n* = 29; *M* = Mean,

*SD* = Standard Deviation; **p* < 0.05, ***p* < 0.01.

Table S3

| *a) Main effect of group (BPD, HC)* | | | | | | |  |  |  |  |  |  |
| --- | --- | --- | --- | --- | --- | --- | --- | --- | --- | --- | --- | --- |
| **label** |  |  | | | ***p_PWE_*_-corr_** | | ***k*_E_** | **F** |  | **MNI (x)** | **MNI (y)** | **MNI (z)** |
| brain region | | | hemisphere | | peak | | cluster |  |  |  |  |  |
| **ACC** |  | | | **L** | | **0.012** | **233** | **22.27** |  | **-12** | **39** | **22** |
| medial frontal gyrus | | | | L | |  | 75 | 19.23 |  | -14 | 46 | 6 |
| subgyral |  | | | L | |  | 8 | 15.53 |  | -10 | 42 | 12 |
| **superior frontal gyrus** | | | | **R** | | **0.004** | **283** | **18.80** |  | **26** | **57** | **15** |
| middle frontal gyrus | | | | R | |  | 67 | 18.35 |  | 15 | 69 | 16 |
| **ACC** |  | | | **R** | | **0.001** | **366** | **17.50** |  | **12** | **40** | **3** |
| medial frontal gyrus | | | | R | |  | 89 | 17.00 |  | 15 | 42 | -4 |
| subgyral |  | | | R | |  | 66 | 13.53 |  | 0 | 46 | -12 |
| *Note.* Height threshold: F=11.17, p= 0.001; Degrees of freedom: (1.0, 193.0); Extent threshold: k=233 voxels; FWEc: 233. | | | | | | | | | | | | |
|  |  |  | | | |  |  |  |  |  |  |  |
| *b) Main effect ball tossing condition (control, inclusion, exclusion)* | | | | | | | | | | |  |  |
| **label** |  | |  | | ***p_PWE_*_-corr_** | | ***k*_E_** | **F** |  | **MNI (x)** | **MNI (y)** | **MNI (z)** |
| brain region | | | hemisphere | | peak | | cluster |  |  |  |  |  |
| **middle frontal gyrus** | | | | **L** | **<0.001** | | **3017** | **41.12** |  | **-48** | **15** | **4.5** |
| superior frontal gyrus | | | | L |  | | 1786 | 27.38 |  | -30 | -9 | 57 |
| inferior frontal gyrus | | | | L |  | | 1759 | 25.33 |  | -24 | 50 | 26 |
| **inferior parietal lobe** | | | | **L** | **<0.001** | | **1675** | **39.35** |  | **-46** | **-54** | **44** |
| supramarginal gyrus | | | | L |  | | 1164 | 27.36 |  | -48 | -48 | 34 |
| angular gyrus | | | | L |  | | 240 | 27.25 |  | -54 | -56 | 27 |
| **Cerebellum Declive** | | | | **R** | **<0.001** | | **830** | **34.94** |  | **32** | **-81** | **-33** |
|  | Uvula | | | R |  | | 320 | 30.54 |  | 20 | -80 | -26 |
|  | Tuba | | | R |  | | 150 | 25.32 |  | 38 | -75 | -32 |
| **middle temporal gyrus** | | | | **L** | **<0.001** | | **722** | **31.51** |  | **-42** | **-40** | **-8** |
| superior temporal gyrus | | | | L |  | | 231 | 29.44 |  | -58 | -39 | -6 |
| fusiform gyrus | | | | L |  | | 3 | 20.12 |  | -60 | -22 | 0 |
| **medial frontal gyrus** | | | | **L** | **<0.001** | | **369** | **22.08** |  | **-6** | **46** | **6** |
| anterior cingulate | | | | L |  | | 315 | 17.99 |  | -3 | 50 | -4 |
| **post central gyrus** | | | | **R** | **<0.001** | | **387** | **21.26** |  | **54** | **-34** | **52** |
| inferior parietal lobule | | | | R |  | | 147 | 15.89 |  | 57 | -24 | 34 |
| precentral gyrus | | | | R |  | | 7 | 14.95 |  | 56 | -33 | 42 |
| **lingual gyrus** | | | | **L** | **0.001** | | **207** | **20.46** |  | **-18** | **-88** | **-4** |
| cuneus |  | | | L |  | | 124 | 11.07 |  | -12 | -96 | 3 |
| **inferior frontal gyrus** | | | | **R** | **<0.001** | | **210** | **19.76** |  | **39** | **14** | **22** |
| middle frontal gyrus | | | | R |  | | 117 | 12.17 |  | 48 | 9 | 32 |
| precentral gyrus | | | | R |  | | 10 | 11.21 |  | 42 | -3 | 24 |
| **middle temporal gyrus** | | | | **R** | **<0.001** | | **924** | **19.68** |  | **32** | **-60** | **-9** |
| superior temporal gyrus | | | | R |  | | 174 | 17.27 |  | 34 | -52 | -14 |
| precuneus |  | | | R |  | | 166 | 15.62 |  | 36 | -68 | 20 |
| **posterior cingulate** | | | | **R** | **0.002** | | **42** | **17.64** |  | **18** | **-58** | **26** |
| precuneus |  | | | R |  | | 24 | 12.19 |  | 15 | -57 | 16 |
| **postcentral gyrus** | | | | **R** | **<0.001** | | **367** | **17.31** |  | **-22** | **-40** | **69** |
| inferior parietal lobule | | | | R |  | | 37 | 15.43 |  | -32 | -39 | 64 |
| superior parietal lobule | | | | R |  | | 4 | 10.49 |  | -39 | -45 | 58 |
| **middle frontal gyrus** | | | | **R** | **<0.001** | | **565** | **15.49** |  | **39** | **8** | **40** |
| inferior frontal gyrus | | | | R |  | | 46 | 14.28 |  | 50 | 10 | 45 |
| superior frontal gyrus | | | | R |  | | 22 | 13.84 |  | 20 | 10 | 48 |
| **inferior parietal lobule** | | | | **R** | **0.001** | | **181** | **11.49** |  | **33** | **-46** | **45** |
| precuneus |  | | | R |  | | 26 | 10.56 |  | 45 | -48 | 46 |
| *Note.* Height threshold: F=7.16, p= 0.001; Degrees of freedom: (1.0, 193.0); Extent threshold: k=273 voxels, FWEc: 273. | | | | | | | | | | | | |
|  | | | | | | |  |  | | | | |
| *c) Interaction effect of group (BPD,HC) and ball tossing condition (control, inclusion, exclusion)* | | | | | | | | | | | |  |
| **label** |  | |  | | ***p_PWE_*_-corr_** | | ***k*_E_** | **F** |  | **MNI (x)** | **MNI (y)** | **MNI (z)** |
| brain region | | | hemisphere | | peak | | cluster |  |  |  |  |  |
| **anterior cingulate** | | | | **L** | **<0.001** | | **1390** | **24.58** |  | **-12** | **38** | **21** |
| medial frontal gyrus | | | | R |  | |  | 15.66 |  | 6 | 52 | 10 |
| superior frontal gyrus | | | | L |  | |  | 13.48 |  | -10 | 44 | 4 |
| **superior frontal gyrus** | | | | **L** | **0.001** | | **339** | **18.71** |  | **-20** | **46** | **34** |
| middle frontal gyrus | | | | L |  | |  | 15.66 |  | -22 | 50 | 27 |
| medial frontal gyrus | | | | L |  | |  | 9.97 |  | -16 | 28 | 40 |
| **precuneus** | | | | **R** | **0.004** | | **255** | **18.57** |  | **9** | **-45** | **72** |
| postcentral gyrus | | | | R |  | |  | 9.54 |  | 14 | -36 | 64 |
| paracentral lobule | | | | R |  | |  | 8.49 |  | 22 | -36 | 66 |
| **medial frontal gyrus** | | | | **R** | **<0.001** | | **535** | **18.46** |  | **12** | **26** | **46** |
| ACC | | | | R |  | |  | 12.76 |  | 4 | 24 | 36 |
| superior frontal gyrus | | | | R |  | |  | 12.34 |  | 8 | 40 | 28 |
| **superior temporal gyrus** | | | | **R** | **<0.001** | | **468** | **15.14** |  | **46** | **-21** | **-2** |
| middle temporal gyrus | | | | R |  | |  | 11.42 |  | 33 | -24 | 6 |
| posterior insula | | | | R |  | |  | 10.72 |  | 57 | -22 | 14 |
| **superior frontal gyrus** | | | | **L** | **0.005** | | **240** | **13.10** |  | **-22** | **52** | **9** |
| medial frontal gyrus | | | | L |  | |  | 12.31 |  | -20 | 62 | 18 |
| middle frontal gyrus | | | | L |  | |  | 10.26 |  | -10 | 58 | 16 |
| **cerebellum** | **Uvula** | | | **L** | **0.006** | | **238** | **12.30** |  | **-10** | **-54** | **-38** |
|  | Nodule | | | L |  | |  | 11.90 |  | -8 | -66 | -42 |
|  | **Culmen** | | | **R** | **0.013** | | **205** | **11.97** |  | **22** | **-51** | **-33** |
|  | Nodule | | | R |  | |  | 11.47 |  | 16 | -60 | -33 |
| *Note.* Height threshold: F=7.16, p=0.001; Degrees of freedom: (2.0, 193.0); Extent threshold: k=205 voxels, FWEc: 205. | | | | | | | | | | | | |
|  |  |  | | | |  |  |  |  |  |  |  |

| *d) T-test (BPD > HC) of exclusion contrast (exclusion condition – inclusion condition)* | | | | | | | | | | | | |
| --- | --- | --- | --- | --- | --- | --- | --- | --- | --- | --- | --- | --- |
| **label** |  | | |  | | ***p_PWE_*_-corr_** | ***k*_E_** |  | **mean T** | **MNI (x)** | **MNI (y)** | **MNI (z)** |
| brain region | | | hemisphere | | | peak | cluster |  | cluster |  |  |  |
| **anterior cingulate** | | | | | **L** | **0.004** | **208** |  | **4.93** | **-14** | **46** | **6** |
| medial frontal gyrus | | | | | L |  | 113 |  | 4.70 | -12 | 39 | 22 |
| **superior frontal gyrus** | | | | | **R** | **<0.001** | **256** |  | **4.73** | **26** | **57** | **15** |
| medial frontal gyrus | | | | | R |  | 135 |  | 4.52 | 16 | 60 | 15 |
| middle frontal gyrus | | | | | R |  | 84 |  | 4.14 | -8 | 60 | 8 |
| **anterior cingulate** | | | | | **R** | **<0.001** | **289** |  | **4.62** | **8** | **38** | **0** |
| medial frontal gyrus | | | | | R |  | 156 |  | 4.45 | 12 | 46 | 3 |
| *Note.* Height threshold: F=3.23 p= 0.001; Degrees of freedom: (1.0, 193.0); Extent threshold: k=336 voxels, FWEc: 336. | | | | | | | | | | | | |
|  |  |  | | | |  |  |  |  |  |  |  |
| *e) Effect of NA as a covariate of interest* | | | | | | |  |  |  |  |  |  |
| **label** |  |  | | | | ***p_PWE_*_-corr_** | ***k*_E_** |  | **mean T** | **MNI (x)** | **MNI (y)** | **MNI (z)** |
| brain region | | hemisphere | | | | peak | cluster |  | cluster |  |  |  |
| **anterior cingulate** | | | | | **R** | **<0.001** | **284** |  | **4.32** | **14** | **44** | **-8** |
| medial frontal gyrus | | | | | R |  | 63 |  | 4.32 | -9 | 28 | -6 |
| superior frontal gyrus | | | | | R |  | 8 |  | 4.29 | 8 | 38 | 0 |
| *Note.* Height threshold: T=3.13, p= 0.001; Degrees of freedom: (1.0, 193.0); Extent threshold: k=205 voxels, FWEc: 482; NA= negative affective state. | | | | | | | | | | | | |

**Figures**

*Figure S1a*. Pre-scan negative affective state (NA): Distribution of sum-scores of the subscale NA of Positive and Negative Affect Schedule (Krohne, Egloff, Kohlmann, & Tausch, 1996), HC=healthy control group, BPD= Borderline Personality Disorder group.

*Figure S1b*. Post-scan negative affective state (NA): Distribution of sum-scores of the subscale NA of Positive and Negative Affect Schedule (Krohne, Egloff, Kohlmann, & Tausch, 1996), HC=healthy control group, BPD= Borderline Personality Disorder group.
